# Progressive multifocal leukoencephalopathy in Finland: a cross-sectional registry study

**DOI:** 10.1007/s00415-018-09167-y

**Published:** 2019-01-05

**Authors:** Jussi O. T. Sipilä, Merja Soilu-Hänninen, Päivi Rautava, Ville Kytö

**Affiliations:** 1 Department of Neurology, Siun sote North Karelia Central Hospital, Tikkamäentie 16, 80210 Joensuu, Finland; 2 Division of Clinical Neurosciences, Turku University Hospital, Turku, Finland; 3 Department of Neurology, University of Turku, Turku, Finland; 4 Department of Public Health, University of Turku and Turku Clinical Research Centre, Turku University Hospital, Turku, Finland; 5 Heart Center, Turku University Hospital, Turku, Finland; 6 Research Center of Applied and Preventive Cardiovascular Medicine, University of Turku, Turku, Finland

**Keywords:** Complication, Epidemiology, Incidence, Infectious disease, Mortality, Neuroimmunology, Predisposing factors, Therapy

## Abstract

**Objective:**

To investigate if progressive multifocal leucoencephalopathy (PML) incidence has increased in Finland like in the neighbouring Sweden.

**Methods:**

National administrative registries were searched for all PML admissions aged 16 years or more in 2004–2014 on all neurological and internal medicine wards in Finland. The mortality data of the patients was extracted from the national causes of death registry. National level data on annual predisposing drug use was obtained from the national pharmaceutical authority.

**Results:**

We identified 35 PML cases (57% male) with a peak in 2010–2011 that amounted to 49% of all cases. The annual incidence for the entire study period was 0.072/100,000 person-years (95% CI 0.050–0.10) with no temporal trend (*p* = 0.18). Mean age was 57 years (22–88 years) with no sex difference (*p* = 0.42). Neoplasms (60%), HIV infection (17%) and systemic connective tissue disorders (CTD, 14%) were the most common predisposing conditions. MS was recorded in three cases (9%). The national level use of drugs that predispose to PML increased during the study period, with the exceptions of alemtuzumab and fludarabine. Overall survival was 85% at 90 days, 79% at 1 year, and 66% at 5 years. Survival was worst in patients with malignancy and best in patients with CTD.

**Conclusions:**

PML most often occurs in patients with malignancies and patients with HIV or CTD cover a third. PML incidence in Finland is lower than in Sweden and shows no temporal trend despite increasing use of predisposing drugs. Mortality after PML varies according to the predisposing condition.

**Electronic supplementary material:**

The online version of this article (10.1007/s00415-018-09167-y) contains supplementary material, which is available to authorized users.

## Introduction

Progressive multifocal leucoencephalopathy (PML) is a rare opportunistic infection of the central nervous system, caused by John Cunningham Virus (JCV) activation in an immunocompromised patient. PML varies in severity from asymptomatic to lethal and therapeutic options are limited [1–3]. Although initially described in patients with underlying B-cell lymphoproliferative disorders, PML was long associated mainly with HIV/AIDS patients in whom combination antiretroviral therapy (cART) has now decreased this risk [3, 4].

In 2000–2008, PML was very rare in non-HIV patient groups [5]. More recently, monoclonal antibodies (MABs) that increase the risk of PML have been increasingly used to treat diseases such as malignancies and autoimmune diseases affecting diverse organ systems [1, 6]. In Sweden, PML incidence was 0.11/100,000 person-years in 2011–2013, a fourfold increase from the long preceding stable level and coinciding with prior use of MAB treatment [7]. Human stem cell transplantation (HSCT) and chemotherapy also increase the risk of PML [3, 8].

PML may also ensue from treating multiple sclerosis (MS) with natalizumab, dimethyl fumarate, fingolimod or ocrelizumab [3]. Of these, the highest risk is associated with natalizumab, the benefits of which patients, neurologists, scientists and the industry may overestimate and PML risk underestimate [2, 9–13]. In Finland, the use of disease modifying treatments (DMTs) has increased and MS-hospitalizations markedly declined from 2004 to 2014 but the proportion of MS admissions with an infection as the primary diagnosis increased, suggesting a trade-off between benefits and harms [14]. Two Finnish MS patients have been reported to have had PML (both with natalizumab, which has 300–400 users) but national PML data is not available.

## Materials and methods

### Data collection

All patients, at least 16 years of age, treated for PML (ICD-10 code A81.2) in Finland (with 4,515,838 persons at least 16 years of age at the end of 2014) in 2004–2014 were identified from the national inpatient registry Care Registry for Health Care (CRHC), maintained by the National Institute for Health and Welfare (THL). CRHC is a database to which all hospitals in Finland are obliged to report all ward discharges. The search included all neurology, internal medicine and surgery wards (including all internal medicine and surgical subspecialty wards) of the five university hospitals and 39 other hospitals on mainland Finland. Predisposing diagnoses (primary diagnosis and auxiliary diagnoses 1 and 2 were included) were analysed. Cases with no recorded diagnosis known to predispose to PML were excluded. Date of the first hospital admission was used as the index date. For geographical analysis, the university hospital expert responsibility area that the patient was treated in was identified. Annual population data were obtained from the national authority, Statistics Finland, which also provided data on patient deaths from the national, mandatory cause of death registry, which was accessed for data extending up to the end of the year 2016. Data on the annual national use of the most important drugs that predispose to PML and were available in Finland (natalizumab, rituximab, infliximab, alemtuzumab, fingolimod, fludarabine, mycophenolate, leflunomide) were obtained from Finnish Medicines Agency Fimea, the national pharmaceutical authority.

### Statistical methods

Shapiro–Wilk test was used to assess the distribution of age and, subsequently, the independent samples *t* test to assess differences between sexes. Poisson regression was used for analysis of count data and Kaplan–Meier method for survival analysis. Confidence intervals for incidence were calculated with Poisson assumption. In the Poisson analysis of incidence trend, Pearson Chi square was used as the scale parameter method because of overdispersion. Statistical significance was considered to be presented by a *p* value < 0.05. Analyses were conducted using SAS System for Windows, version 9.4 (SAS Institute Inc., Cary, NC, USA) or IBM SPSS Statistics for Windows, Version 24.0 (Armonk, NY: IBM Corp).

## Results

**Fig. 1:**
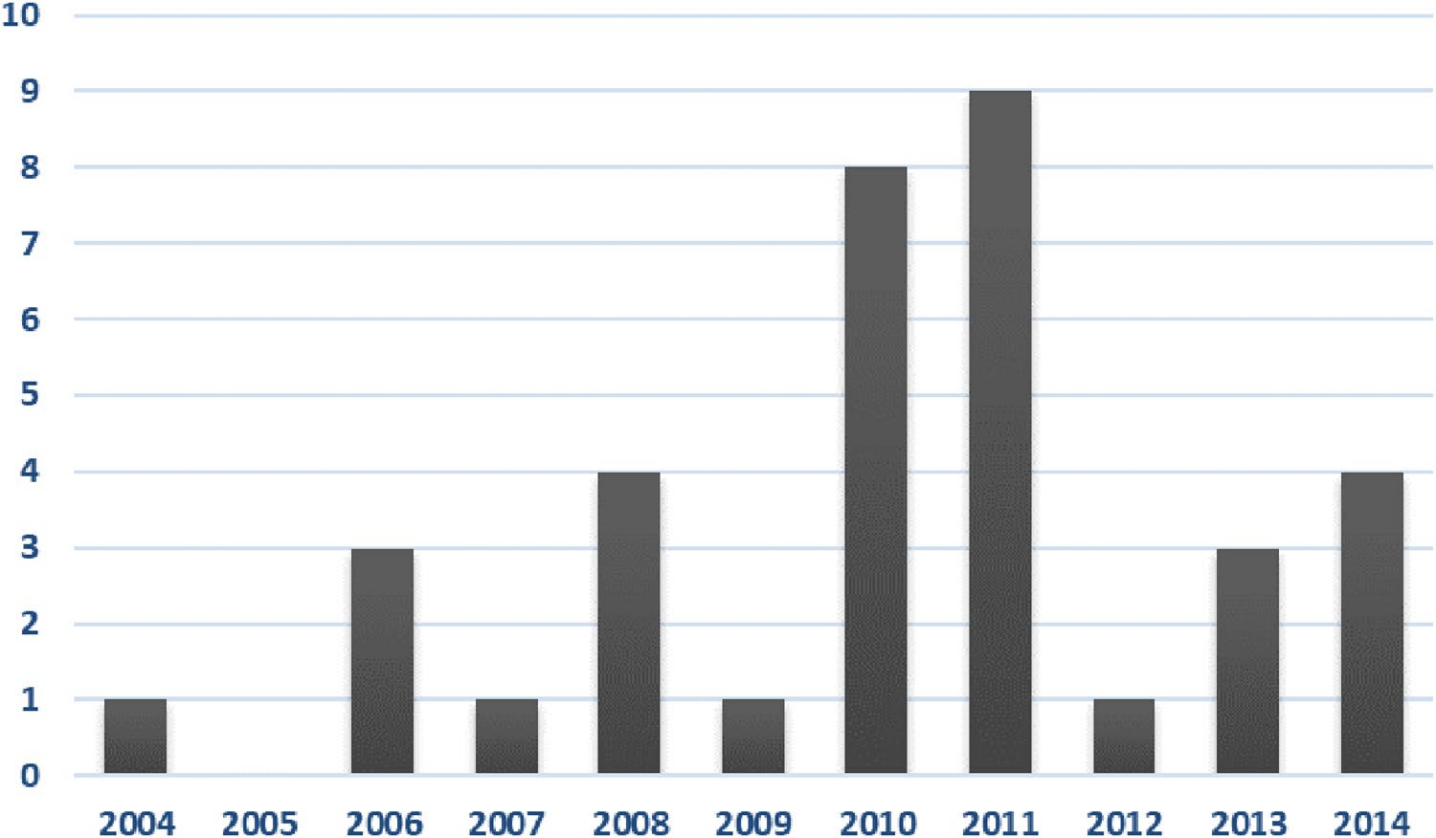
The annual number of PML-admissions in Finland

We identified 35 persons (57% male) treated for PML with a peak in 2010–2011 that amounted to 49% of all cases (Fig. 1). The annual incidence for the entire study period was 0.072/100,000 person-years (95% CI 0.050–0.10). In 2004–2009 the incidence was 0.038/100,000 person-years (95% CI 0.012–0.071) and in 2012–2014 0.059/100,000 person-years (95% CI 0.026–0.12). Poisson loglinear analysis showed no trend in annual incidence rates (*p* = 0.18). Cases had been recorded in nine out of the 20 hospital districts and were observed in every university hospital’s expert responsibility and teaching area (supplementary figure). Nearly half (16) of the cases had been treated in the Helsinki University Hospital teaching area and the same number (16) spread between Tampere, Turku and Oulu university hospitals’ areas. No cases were recorded for Kuopio university hospital and only two in the central hospitals in its special responsibility and teaching area. One patient had been treated in three areas during uninterrupted, sequential admissions and was left out of the geographical analysis.

**Fig. 2:**
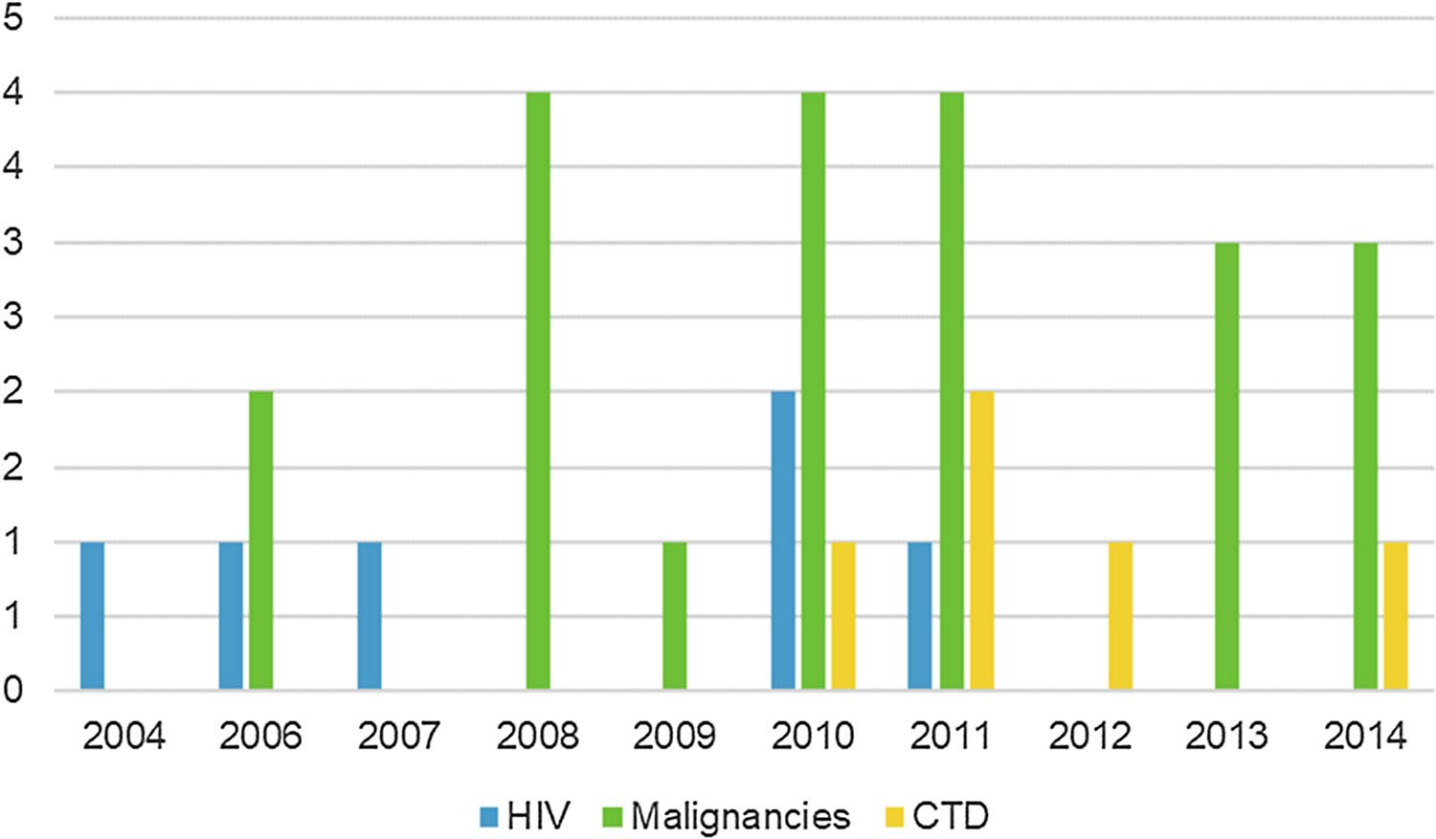
The annual frequencies of the three major predisposing diagnostic categories in PML admissions

Mean patient age was 57 years (SD 17.7; range 22–88 years) with no difference between sexes (*p* = 0.42). The majority (71%) of cases were treated on neurology wards. Predisposing conditions were most often neoplasms (60%; ICD-10 groups C and D) with HIV infection (17%) and Systemic connective tissue disorders (14%; CTDs, ICD-10 group M) trailing. The connective tissue disorders consisted of 2 cases of systemic lupus, 1 of Behçet’s disease, 1 of dermatomyositis and 1 of polyarteritis nodosa. None of the PML cases observed after 2011 was associated with HIV and none observed before 2010 was associated with a CTD while cases associated with malignancies were spread across the study period (Fig. 2). MS was implicated in three cases (9%) which all occurred in 2010–2011.

**Fig. 3:**
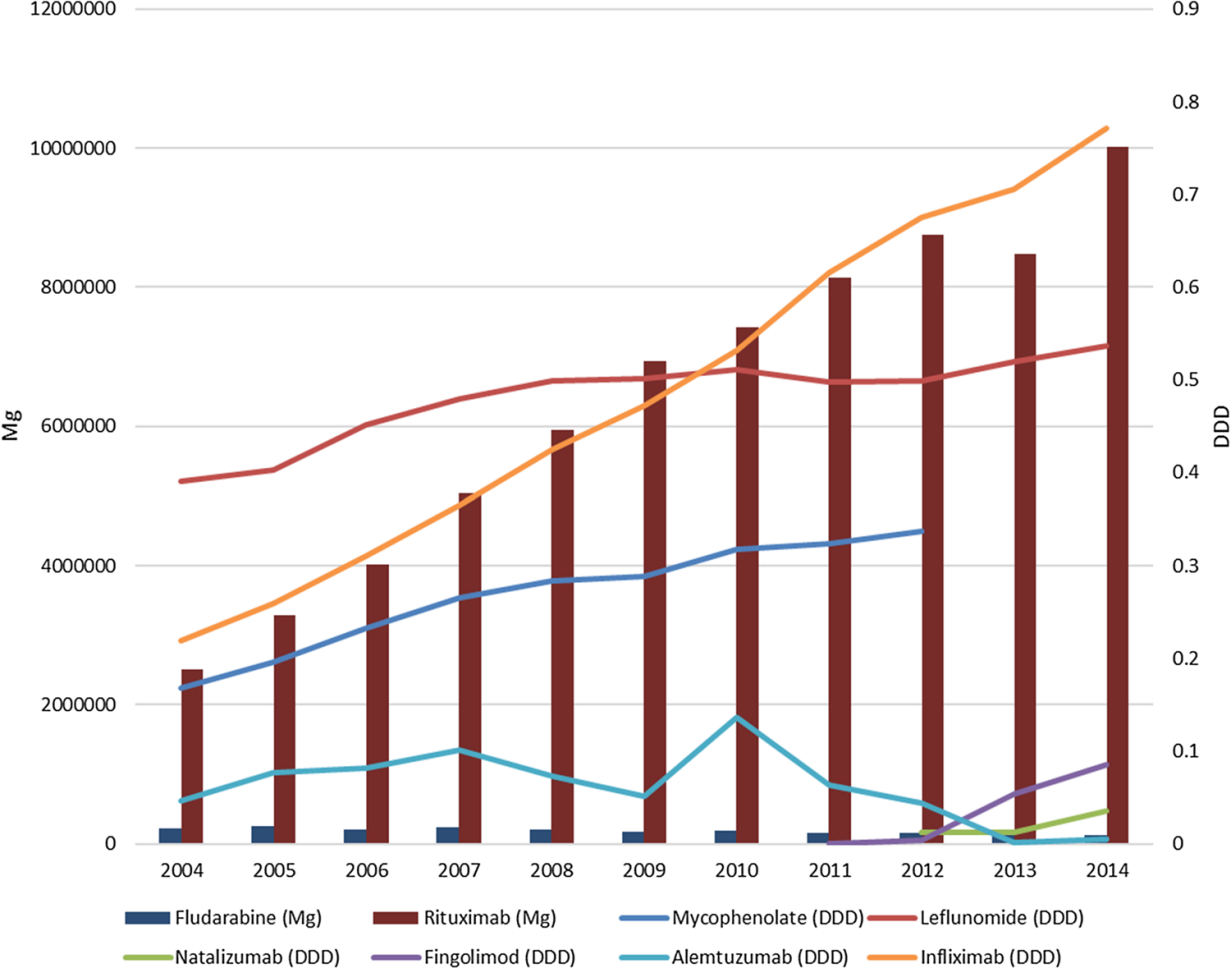
The national level annual use of drugs that predispose to PML during the study period

The national level use of drugs that predispose to PML increased during the study period with the exceptions of alemtuzumab and fludarabine (Fig. 3).

**Fig. 4:**
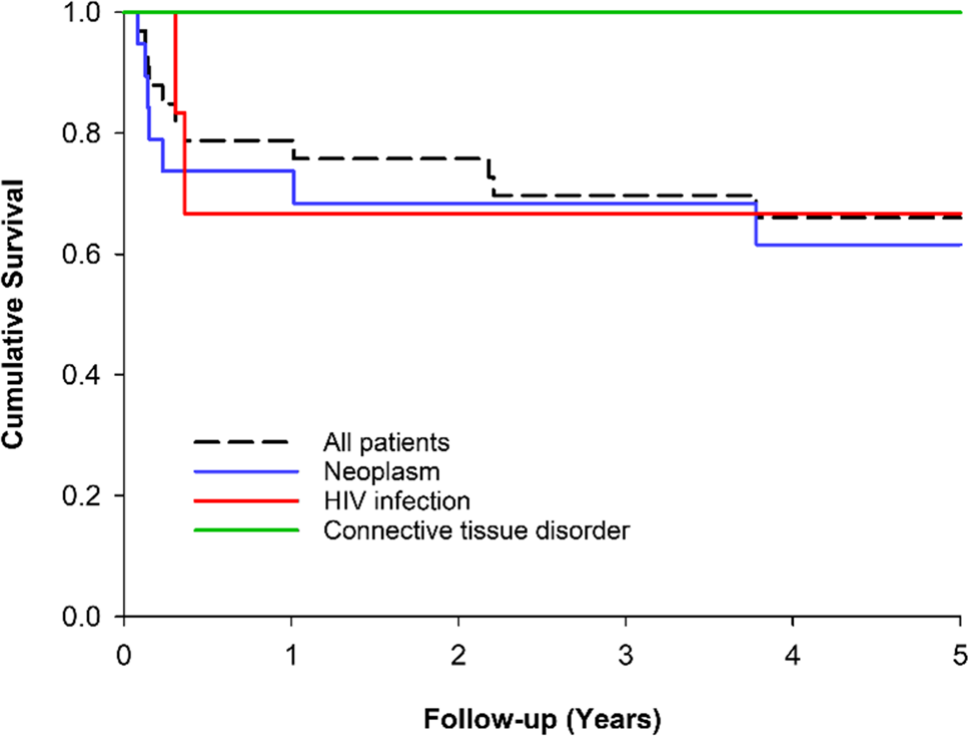
Five-year survival in PML patients according to the predisposing condition. Only three largest predisposing groups are presented separately. However, patients with multiple sclerosis as the predisposing factor are included in the analysis of all patients

Two (6%) of the 35 patients died while in hospital, both men with a primary diagnosis of HIV (27 and 36 years of age). Overall survival was 85% at 90 days, 79% at 1 year and 66% at 5 years (long-term survival data was available for 33 patients). The survival prognosis was worst in cases of PML associated with a malignancy and best in cases associated with connective tissue disease (in which survival was 100% at 5 years) (Fig. 4).

## Discussion

In this nationwide study from 2004 to 2014 we found a low adult-onset incidence of PML without a convincing temporal trend in Finland. The majority of the cases were associated with malignancies. The proportions of patients with HIV or CTDs were modest and, despite increasing DMT use, the proportion of MS was less than a tenth. Compared to neighbouring Sweden, PML incidence is lower in Finland and the increasing trend is missing even though predisposing drug use and HSCT rates have increased [7, 16].

In nearly two-thirds of cases, we identified a neoplasm as the predisposing condition with nearly all the rest associated with either HIV or systemic autoimmune diseases. The proportion of MS as the predisposing diagnosis was under 10%. These findings are similar to those reported for Sweden [7]. However, the overall incidence we report is lower than that in Sweden. This might result from methodological differences in case ascertainment and age limits between the studies. However, this is unlikely since registry studies that do not include case review usually report higher figures than those found by the retrospective studies that review medical records [15]. Indeed, the difference may be a real one since the Swedish study reported that the incidence of PML increased from a stable average of 0.026/100,000 person-years in 1988–2010 to 0.11/100,000 in 2011–2013, whereas our data, that extended up to 2014, revealed no such tendency. Interestingly, the Swedish rate in 1988–2010 is within the 95% confidence intervals we observed in Finland when excluding the aberrant peak of 2010–2011 in our data. These differences between studies after the year 2010 are unlikely to be caused by differences in predisposing drug use, since our data showed that their national use, especially of rituximab and infliximab, increased during the study period and appeared unrelated to PML incidence. MABs with the highest risk of PML are rituximab (which has been associated with PML when used in other diseases but not MS) and natalizumab [6], the use of both which increased in Finland during the study period. Moreover, HSCT is very actively used in Finland and its frequency has increased from 2004 to 2014 [16]. It is unclear why PML incidence has not increased accordingly.

The aberrant PML case peak of 2010–2011 in our data might be associated with changes in JCV testing methods since this peak was observed for all predisposing diagnoses. No predisposing drug showed a trend associated with this, save for alemtuzumab, the use of which peaked in 2010 and trailed off towards the end of the study period (it received an indication for MS in Finland only at the end of 2013). All PML cases associated with MS observed in our study occurred during these years and two of these cases have been published previously (one as a case report [17] and one as an oral presentation at a national neuroimmunology meeting) and were associated with natalizumab. Interestingly, all PML cases associated with CTDs were observed after 2009, suggesting that changing treatment strategies of these diseases should be looked at in particular. On the other hand, no PML cases associated with HIV were recorded after 2011. This is particularly interesting because the Helsinki University hospital HIV registry, which contains data on half of HIV patients in Finland, shows a high rate of medication adherence (> 97%) and HIV-PCR < 50 viral suppression (> 96%) (Anna Hanttu, personal communication), suggesting that efficient cART treatment may be implicated in the decline and disappearance of PML cases in Finnish HIV patients but further studies are needed to confirm this.

We observed three cases of PML associated with MS. Unfortunately, we do not have data on their treatment regimens, but epidemiological aspects may be discussed. Natalizumab was introduced in Finland in 2006 and has reached a level of 300–400 patients receiving the drug. Fingolimod, introduced in 2012, had reached a level of 591 users by the end of 2014 when dimethyl fumarate and ocrelizumab (for which all PML cases observed so far have occurred in patients who have discontinued other medications shortly before (Natalizumab or Fingolimod) and have, therefore, all been classified as carry-over cases) were not yet available in Finland. Considering the reported risk rates of natalizumab- and fingolimod-associated PML [18, 19], the number of cases in our data seems in line with previous research and indicates no new cause for concern.

Two-years from PML, only 26% of the patients were alive in Sweden, whereas 66% of patients were alive 5 years after the diagnosis in our study [7]. This discrepancy might indicate differences in treatment protocols and patient selection for aggressive immunosuppressive therapies between the countries. This might particularly concern patients with malignancies since in the Swedish study the mortality hazard of these patients appeared to be markedly higher compared to HIV, whereas in our data it was quite similar between these patient groups. Both studies found higher mortality in patients whose predisposing condition was a malignancy or HIV compared to those with an autoimmune disease/CTD. In HIV patients with PML, we observed a 3-year survival of 66% that is similar to recent results from Paris, France and Madrid, Spain [4, 20]. The mortality rate of PML patients with a malignancy in our data is relatively low and may be associated with the high frequency of HSCT use in Finland as this treatment is associated with favourable PML outcome compared to chemotherapy [8, 21].

Our study relies on retrospective registry data with no chart verification and may, therefore, overestimate the incidence of PML since the Swedish study reported that over 50% of the registry cases did not meet consensus criteria for PML [7]. On the other hand, they reported that 82% of the cases recorded in neurology departments were accurate and 71% of our data is from neurology wards which suggests reasonable accuracy, whereas in the Swedish study only 43% of the originally identified 250 cases were from neurology wards which means that the remaining 57% of the data, with a diagnostic accuracy of 31% was the major source of false positives. Furthermore, our data does not include oncological wards and therefore some cases may have been missed. However, this is not likely to markedly bias our study since the Swedish study found only 2% of their confirmed cases on oncology wards and already the majority of cases in our data were associated with malignancies indicating that they are treated primarily on the wards we have included. This we also know from clinical practice. In all, our search included the wards where the Swedish study found 96% of their confirmed cases. Furthermore, identifying any false positives in our data would only increase the difference in incidences between the studies and thereby only strengthen our conclusions as it appears unlikely that a possible overrepresentation of PML in our data would be biased in a way that would affect our survey of temporal trend over a decade.

Our data unfortunately does not include any individual level information on drugs that may have predisposed to PML or those used to treat it and other outcomes than mortality remain uncertain. On the other hand, our study covers all PML cases in Finland over more than a decade and the registry has been found to be reliable [22]. We also checked the accuracy of our CRHC data against the Helsinki University hospital HIV registry and both registries reported two cases of PML associated with HIV in Helsinki University hospital (Anna Hanttu, personal communication) suggesting good reliability. A regional survey of PML in the Helsinki and Uusimaa region found a slightly higher annual incidence than our data but the survey extended over a longer period, up to year 2016 (Marge Kartau, personal communication). Similar to our data, malignancies and HIV infection comprised 77% of the predisposing conditions observed in the survey. Our data, therefore, appears reliable.

In conclusion, the majority of PML cases in Finland were observed in patients with malignancies and patients diagnosed with HIV or a connective tissue disorder covered nearly all the rest. The incidence rate was lower than in neighbouring Sweden and, contrary to the Swedish data, showed no temporal trend despite increases in the use of predisposing drugs use on the national level. Further studies on the causes of these differences seem warranted.

## Electronic supplementary material

Below is the link to the electronic supplementary material.

Supplementary figure. The number of PML cases in each university hospitals’ expert responsibility and teaching area (which are distinguished by different shades of grey). (PNG 76 KB)
