# Supplementary figures and images for: Progressive multifocal leukoencephalopathy in Finland: a cross-sectional registry study
Source: J Neurol. 2019 Jan 5;266(2):515–21. doi: 10.1007/s00415-018-09167-y (PMC6373365; doi:10.1007/s00415-018-09167-y)

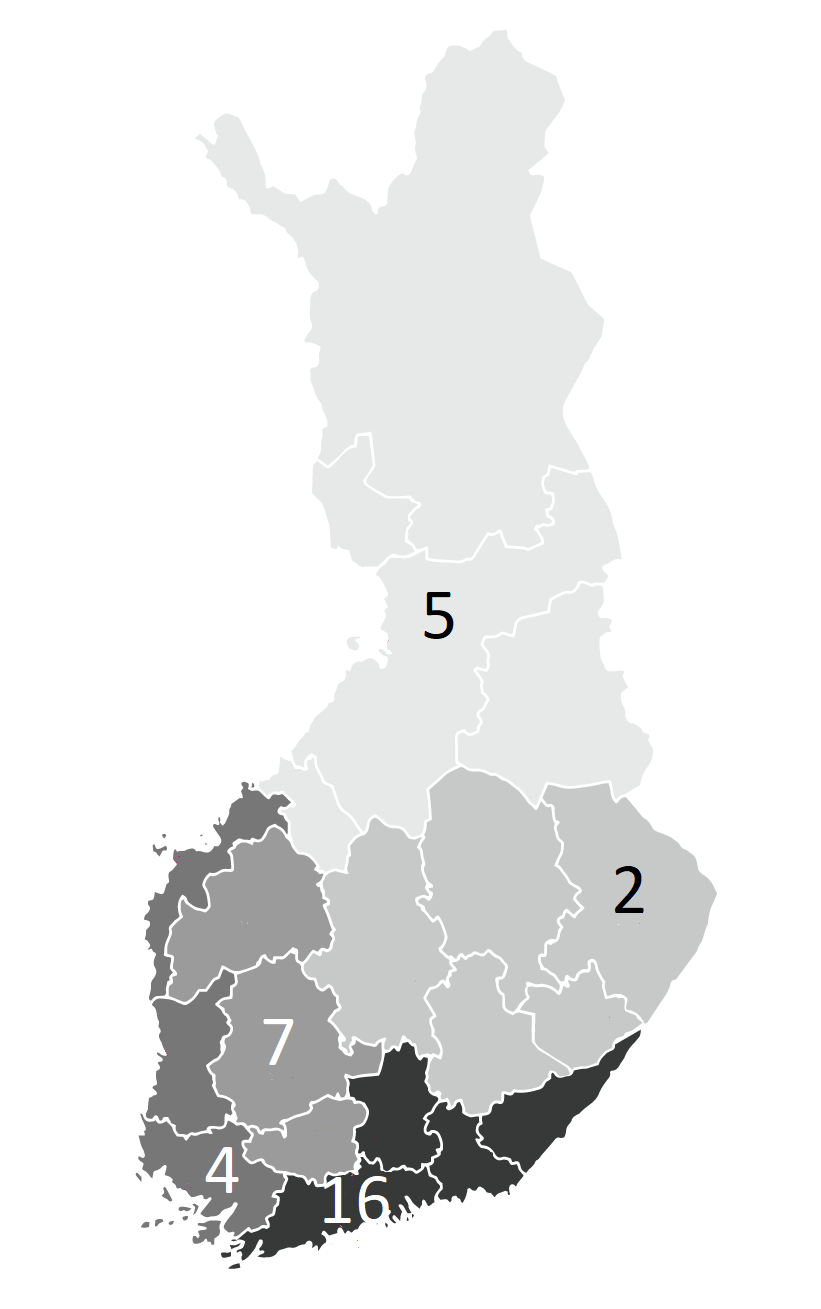

Supplement: Supplementary file 1 — Supplementary figure. The number of PML cases in each university hospitals’ expert responsibility and teaching area (which are distinguished by different shades of grey). (PNG 76 KB) [file 415_2018_9167_MOESM1_ESM.png]
